# Assessing data availability of NCD prevention and control in six ASEAN countries based on WHO global monitoring framework and the progress monitor indicators

**DOI:** 10.1186/s12889-023-15165-1

**Published:** 2023-02-07

**Authors:** Bundit Sornpaisarn, Yuriko Limmade, Supa Pengpid, Isareethika Jayasvasti, Pheak Chhoun, Vathsana Somphet, Feisul Idzwan Mustapha, Kyaw Kan Kaung, Chanatip Chailek, Tran Quoc Bao, Jürgen Rehm

**Affiliations:** 1 Institute for Mental Health Policy Research, Centre for Addiction and Mental Health (CAMH), 33 Ursula Franklin Street, M5S 2S1 Toronto, ON Canada; 2 Dalla Lana School of Public Health, University of Toronto, 6th Floor, 155 College Street, M5T 3M7 Toronto, ON Canada; 3 Faculty of Public Health, Mahidol University, 420/1 Ratchawithi Road, Thung Phaya Thai, Ratchathewi, 10400 Bangkok, Thailand; 4 Medical Service Department, International SOS, Jl. Pangeran Antasari No. 10, Cipete, 12410 Jakarta, Indonesia; 5 KHANA Center for Population Health Research, #33, street 71, Tonle Bassac, Phnom Penh, Cambodia; 6 Department of Epidemiology and Statistic, University of Health Science, Ban Kaoyot, Samsenthai Rd., Vientiane Capital, Lao PDR; 7 Disease Control Division, Ministry of Health, Putrajaya, Malaysia; 8 Department of Public Health, Ministry of Health and Sports, Naypyidaw, Myanmar; 9 Field Epidemiology Training Program (FETP), Division of Epidemiology, Department of Disease Control, Ministry of Public Health, Mueang Nonthaburi, Thailand; 10 Non-Communicable Diseases Control Division, General Department of Preventive Medicine, Viet Nam Ministry of Health, Hanoi, Viet Nam; 11 Campbell Family Mental Health Research Institute, CAMH, 250 College Street, M5T 1R8 Toronto, ON Canada; 12 Department of Psychiatry, University of Toronto, 8th Floor, 250 College Street, M5T 1R8 Toronto, ON Canada; 13 Institute of Medical Science, University of Toronto, 1 King’s College Circle, M5S 1A8 Toronto, ON Canada; 14 Department of International Health Projects, Institute for Leadership and Health Management, I.M. Sechenov First Moscow State Medical University, Trubetskaya str., 8, b. 2, 119992 Moscow, Russian Federation; 15 Institute of Nutrition, Mahidol University, 999 Phutthamonthon sai 4, 73170 Nakhon Pathom, Thailand

**Keywords:** ASEAN, Data, Global monitoring Framework, NCD, Indicator, Progress Monitoring Indicator, Surveillance

## Abstract

**Background:**

To tackle noncommunicable disease (NCD) burden globally, two sets of NCD surveillance indicators were established by the World Health Organization: 25 Global Monitoring Framework (GMF) indicators and 10 Progress Monitoring Indicators (PMI). This study aims to assess the data availability of these two sets of indicators in six ASEAN countries: Cambodia, Lao PDR, Malaysia, Myanmar, Thailand, and Vietnam.

**Methods:**

As data on policy indicators were straightforward and fully available, we focused on studying 25 non-policy indicators: 23 GMFs and 2 PMIs. Gathering data availability of the target indicators was conducted among NCD surveillance experts from the six selected countries during May-June 2020. Our research team found information regarding whether the country had no data at all, was using WHO estimates, was providing ‘expert judgement’ for the data, or had actual data available for each target indicator. We triangulated their answers with several WHO data sources, including the WHO Health Observatory Database and various WHO Global Reports on health behaviours (tobacco, alcohol, diet, and physical activity) and NCDs. We calculated the percentages of the indicators that need improvement by both indicator category and country.

**Results:**

For all six studied countries, the health-service indicators, based on responses to the facility survey, are the most lacking in data availability (100% of this category’s indicators), followed by the health-service indicators, based on the population survey responses (57%), the mortality and morbidity indicators (50%), the behavioural risk indicators (30%), and the biological risk indicators (7%). The countries that need to improve their NCD surveillance data availability the most are Cambodia (56% of all indicators) and Lao PDR (56%), followed by Malaysia (36%), Vietnam (36%), Myanmar (32%), and Thailand (28%).

**Conclusion:**

Some of the non-policy GMF and PMI indicators lacked data among the six studied countries. To achieve the global NCDs targets, in the long run, the six countries should collect their own data for all indicators and begin to invest in and implement the facility survey and the population survey to track NCDs-related health services improvements once they have implemented the behavioural and biological Health Risks Population Survey in their countries.

**Supplementary Information:**

The online version contains supplementary material available at 10.1186/s12889-023-15165-1.

## Background

Noncommunicable diseases (NCDs) are an important global issue. In 2016, an estimated 40.5 million deaths worldwide (71% of the 56.9 million deaths worldwide) were caused by NCDs [1]. Of these, around 15.2 million deaths (38%) occurred in people aged between 30 and 70 years old [1]. An estimated 32.2 million NCD deaths (80%) were attributed to cancers, cardiovascular diseases, chronic respiratory diseases, and diabetes. All other NCDs caused 8.3 million NCD deaths (accounting for 20% of the total) [1]. There were two crucial global declarations that addressed the significance of the prevention and control of NCDs: the 2011 United Nations High-Level Meeting (UNHLM) on the Prevention and Control of NCDs [2], and the United Nations (UN)’s Sustainable Development Goals (SDGs) [3].

After the UNHLM *Political Declaration on the Prevention and Control of Non-communicable Diseases* was adopted in 2011, the WHO developed a GMF to enable tracking of progress in preventing and controlling major NCDs (including cardiovascular diseases, cancers, diabetes, and chronic respiratory diseases) and their shared risk factors (covering smoking, harmful use of alcohol, unhealthy diet, and physical inactivity) [2]. This GMF, consisting of 25 indicators and nine global targets, was adopted by the WHO Member States in 2013 [2]. WHO then published a document entitled ‘NCD Global Monitoring Framework: Indicator Definitions and Specifications’ in 2014, aiming to provide guidance to the Member States on how to correctly measure each of the 25 indicators and monitor their progress over time [2]. This document describes the indicators’ definitions, method(s) of estimation/ calculation, preferred data sources, etc. Included in the GMF indicators are two indicators regarding mortality and morbidity data, eight behavioural risk factors, seven biological risk factors, and eight national system responses [2] (see the Additional file 1 for details of the NCD GMF Framework). Since the nine global targets are overlap with some of these 25 indicators, analyzing the availability of data for them also includes a data availability analysis of the nine global targets. Each of the nine global targets has one or several corresponding key indicators, plus there are some additional indicators. For example, for the first target on mortality and morbidity (see Appendix 1), cancer incidence by type of cancer is listed as additional indicator. These include cancer incidence, the mean proportion of total energy intake from saturated fatty acid, the prevalence of persons consuming less than five total servings (400 grams) of fruit and vegetable per day, the prevalence for raised total cholesterol, access to palliative care, the adoption of national policies that limit saturated fatty acids in the food supply, the availability of human papillomavirus vaccines, policies to reduce the impact on children of marketing of foods and non-alcoholic beverages high in saturated fats, trans fatty acids, or salt, vaccination coverage against hepatitis B virus, and proportion of women between the ages of 30–49 screened for cervical cancer.

Subsequently, in 2015, the WHO published a Technical Note on how it would report on the progress achieved in the implementation of national commitments included in the 2011 UN Political Declaration and the 2014 UN Outcome Document on NCDs to the United Nations General Assembly [4]. This Technical Note outlined a set of 10 Progress Monitor Indicators (PMIs) selected to demonstrate countries’ achievements in implementing the selected national commitments included in the 2014 Outcome Document [4]. Included in these PMIs were four governance and surveillance indicators, four sets of indicators on policies targeting the reduction of the four main NCD risk factors, and two indicators regarding health-service capacities [4].

To showcase global progress on NCD initiatives, the WHO continuously publishes two documents, the *Global Report on NCDs* and the *NCD Progress Monitor*, to track countries’ achievements on indicators pertaining to the GMF, and the PMIs themselves. The Global Report on NCDs provides information addressing the global targets, covering some of the GMF indicators [5, 6]. Covering the information on the Progress Monitor Indicators, the NCDs Progress Monitor reports were published by the WHO in 2015, 2017, and 2020 [4, 7, 8].

The WHO recommends that Member States measure key areas of the NCD epidemic and integrate NCD surveillance data into the national health information systems [9]. To control NCDs in each country effectively, the availability of NCD-related data at the country level is crucial because these data are essential for programme and policy planning, implementation, and evaluation. This study aims to analyze the data availability of the GMF indicators and the PMIs at the country level. Two gaps relating to the tracking of these indicators drive our study. The first gap we wish to address is the lack of data for some indicators at the global level. The WHO reports on the global and country status of the voluntary NCD global targets via two reports, namely the *Global Status Report on NCDs* [5] and the *Noncommunicable Diseases Country Profiles* [6]. The WHO also provides the global and country status of the PMIs through the *NCD Progress Monitor* reports. Thus, the balance of the 25 GMF indicators, beyond the nine voluntary targets and the 10 PMIs, are not reported at the global level. The second gap we wish to address is the lack of data collected for some of these indicators in certain countries which rely instead on estimates made by the WHO [10]. The WHO encourages all Member States to provide information on the status of all relevant indicators they collect to enable a comprehensive reporting of global status even though it is not mandatory for all countries to report on all of the 25 indicators [2]. As a result, the WHO provides country-comparable estimates to inform global values when countries do not have recent data to report for specific indicators [2]. These two deficiencies sparked our interest in assessing the data availability for these NCDs surveillance indicators at the country level, to determine whether there are opportunities to improve the data collection on some specific indicators.

In 2019–2020, Thai Health Promotion Foundation and the WHO initiated a collaborative project to strengthen the NCDs surveillance system for the six selected ASEAN countries: Cambodia, Lao PDR, Malaysia, Myanmar, Thailand, and Vietnam. Hence, the aim of this study, funded by the above-mentioned collaborative project, focused on the analysis of the availability of data for the NCD-related indicators for these six countries specifically. The results may provide guidance as to what should be done to improve the collection of the NCD surveillance data at the country level for these target countries.

## Methods

After initially exploring both the GMF and PMI indicators, we found that all policy-related indicators in both indicator sets are straightforward (based only on the country reporting) and complete, reported in the *Progress Monitor Report* [4, 7, 8]. Hence, our study focused on the data availability on the non-policy indicators of the GMF and PMI indicators.

We used data from several sources to analyze the data availability for the 23 GMF non-policy indicators and two PMIs for the six countries, accounting for 25 indicators in total. First, we used a data-availability collection form to gather the 25-indicators’ data availability at the country level during May-June 2020. The form was filled in by country members of our research team of the ASEAN NCDs Surveillance System Enhancement Project which was funded by the Thai Health Promotion Foundation. These country teams are formed by individuals who work in academic institutions and government ministries related to NCD surveillance. The academics and researchers were selected based on their expertise on NCD prevention and control, their research, and by lectures they provided through their universities or government agencies. The team members from the Ministry of Health are senior personnel from NCDs Prevention and Control departments, divisions, or units. The response options for each indicator included whether the country had no data at all [score of 0], had data but only from the WHO estimates, or were simply ‘expert judgment’ made by the country’s responsible health personnel [score of 1], or had empirical data available at the country level [score of 2]. The reason behind using this simple scoring method was to provide transparency regarding a country’s lack of data. The country team had to provide an example of empirical data for each indicator to confirm their responses. Furthermore, we checked the correctness of the responses provided by the country teams using the following data sources: first, we checked them against data from the WHO secondary-data survey regarding assessing the national capacity to prevent and control NCDs in 2019. This secondary-data survey, which had no human-data involvement, also asked about the empirical data availability of many of the GMF and PM indicators. For example, this capacity-assessment survey asked whether or not the country has a cancer registry. Second, we also checked them against the WHO Health Observatory Database and various WHO Global Reports on health behaviours (tobacco, alcohol, diet, and physical activity) and NCDs. If there were any answers that did not match with the triangulation data, we asked the country team to confirm the existence of data for those indicators. If the country team responded ‘no data at all’ for any indicator, but data was available for that indicator in the relevant WHO report, we would assess the country as having data available through the WHO estimate.

We grouped the indicators into five groups: (i) mortality and morbidity indicators, (ii) behavioural risk indicators, (iii) biological risk indicators, (iv) health-service indicators based on the population survey, and (v) health-service indicators based on the facility survey. See Table 1 for the indicators’ numbers and their data availability status. The mortality and morbidity indicators contained Indicators #1 and #2. The behavioural risk indicators covered Indicators #3–10 and #15–16. Indicators #11–14 and #17 belonged to the biological risk indicators group. The health-service indicators based on the population surveys included Indicators #18 and #20–23, while the indicators based on the facility surveys covered Indicators #19 and #24–25. We then counted the number of indicators that needed data improvement for each country. We counted the indicators for which the country team answered ‘0’ (no data at all) or ‘1’ (either using the WHO estimate or expert judgement at the data), as indicators that needed improvement. Our study created an ‘indicator-country’ rating table to summarize the total number of indicators that needed improvement for each of the countries. For example, in Table 2, in the category of mortality and morbidity, Cambodia is identified as needing data improvement for two indicators, Lao PDR needs improvement on one, Malaysia needs improvement on one, and Vietnam needs improvement on two, whereas both Myanmar and Thailand have their own data for this indicator category and therefore do not need improvement. Thus, the total indicator-country value for the countries that needed improvement in this category was six. We calculated the percentage of the 25 target indicators that needed improvement for each country to see which countries needed to urgently improve their data collection system. Moreover, we also calculated the percentage of the indicator-country that needed improvement for each indicator category to see which indicator categories urgently needed improvement.

**Table 1:** Data availability for 23 non-policy Global Monitoring Framework (GMF) indicators and two health-service indicators of 10 Progress Monitoring Indicators (PMIs) for six selected ASEAN countries

Indicator	Definition of data availability	KH	LA	MY	MM	TH	VN	# of countries needing data improvement
GMF								
1. Premature NCD mortality	- The country has its own data on probability of dying from NCD prematurely.	1	1	1	2	2	1	4
2. Cancer incidence	- The country has its own estimated cancer incidence rate.	1	2	2	2	2	1	2
3. APC	- The country has its own APC estimation	1	1	1	1	2	2	2
4. HED	- The country has its own data on prevalence of heavy episodic drinking	2	2	2	2	2	2	0
5. AUD / AD	- The country has its own data on prevalence of alcohol use disorder / dependence	1	1	1	1	2	1	5
6. PiA – adolescents	- The country has its own data on prevalence of physical inactivity in adolescents	1	1	2	2	2	2	2
7. PiA – adults	- The country has its own data on prevalence of physical inactivity in adults	2	2	2	2	2	2	0
8. Salt intake	- The country has its own data on mean population intake per day in grams	2	1	2	2	2	2	1
9. Tobacco use – adolescents	- The country has its own data on prevalence of tobacco use in adolescents.	2	2	2	2	2	2	0
10. Tobacco use – adults	- The country has its own data on prevalence of tobacco use in adults.	2	2	2	2	2	2	0
11. Raised blood pressure	- The country has its own data on either prevalence of either raised blood pressure or mean blood pressure	2	2	2	2	2	2	0
12. Raised blood glucose / diabetes	- The country has its own data on prevalence of raised blood glucose or diabetes.	2	2	2	2	2	2	0
13. OW/OB – adolescents	- The country has its own data on prevalence of overweight and obesity in adolescents.	1	1	2	2	2	2	2
14. OW/OB – adults	- The country has its own data on prevalence of overweight and obesity in adults.	2	2	2	2	2	2	0
15. Saturated fat	- The country has its own data on mean proportion of total energy intake from saturated fatty acids (SFA).	0	0	0	0	0	0	6
16. Fruit and vegetable	- The country has its own data on prevalence of low fruit and vegetable consumption.	2	2	2	2	2	2	0
17. Cholesterol	- The country has its own data on either prevalence of raised cholesterol or mean cholesterol consumption.	2	2	2	2	2	2	0
18. Drug therapy and counselling – population accessibility	- The country has its own data on percentage of eligible persons (defined as aged 40 years and older with a 10-year cardiovascular disease (CVD) risk > 30%, including those with existing CVD) receiving therapy and counseling (including glycaemic control) to prevent heart attacks and strokes.	2	1	1	2	1	2	3
19. Essential medicines and technologies	- The country has its own data on percentage of public and private primary health care facilities that had all of the essential medicines and technologies.	1	1	1	1	1	1	6
20. Palliative care	- The country has its own data on the availability of palliative care for patients with NCD in the public health system.	1	1	1	1	1	1	6
21. HPV vaccination	- The country has its own data on the HPV vaccine coverage (last dose) in the last calendar year.	1	1	2	0	1	1	5
22. HBV3 vaccination	- The country has its own data on the percentage of one-year old who have received three doses of hepatitis B vaccine in a given year	1	2	2	2	2	2	1
23. Cervical cancer screening	- The country has its own data on the proportion of women aged 30–49 who report they were screened for cervical cancer using any of the following methods: visual inspection with acetic acid/vinegar (VIA), Pap smear and Human Papillomavirus (HPV) test.	1	1	2	2	2	2	2
PMI								
24. National guidelines for NCDs management	- The country has its own data on the percentage of health care facilities that utilize the national guidelines for NCDs management.	1	1	1	1	1	1	6
25. Drug therapy and counselling – provision	- The country has its own data on the proportion of primary health care facilities that are offering cardiovascular risk stratification for the management of patients at high risk for heart attack and stroke.	1	1	1	1	1	1	6

APC: Alcohol Consumption Per Capita, AD: Alcohol Dependence, AUD: Alcohol Use Disorder, GMF: Global Monitoring Framework, HBV3: Hepatitis B Virus – three doses, HED: Heavy Episodic Drinking, HPV: Human Papilloma Virus, KH: Cambodia, LA: Lao PDR, MY: Malaysia, MM: Myanmar, NCD: Non-Communicable Disease, OW/OB: Overweight and Obesity, PiA: Physical Inactivity, PMI: Progress Monitoring Indicator, SDG: Sustainable Development Goal, TH: Thailand, VN: Vietnam

0 indicates no data available, 1 either WHO estimated data (for indicators 1–17) or expert judgment (for indicators 18–25), and 2 data from countries sources such as general population or facility surveys

See Additional file 1 and [2] for the detail name and definition of all indicators shown in Table 1

This study has received an ethics approval exemption from the CAMH Research Ethics Board because it was a documentary study done by six ASEAN country research teams to gather the availability of relevant retrospective data in their countries. The informed consent is not applicable, as no data were collected from respondents outside the research team.

## Results

Table 1 shows the data availability for the 25 NCD surveillance target indicators (23 non-policy GMF indicators and two health service PMI indicators) for the six selected countries (Cambodia, Lao PDR, Myanmar, Malaysia, Thailand, and Vietnam). For example, for the premature NCD mortality indicator (Indicator #1: the unconditional probability of dying between the ages of 30 and 70 years from cardiovascular diseases, cancers, diabetes, or chronic respiratory diseases), two countries had their own data (Myanmar and Thailand) [score of 2] while four countries had no data for this indicator and used WHO estimates instead [score of 1]. Thus, four countries needed data collection improvement for this indicator. See Table 2 for a summary of the data availability by indicator categories, and see Table 1 for further detail on those indicators.

**Table 2:** Data availability of the six selected ASEAN countries for the 25 target indicators, by category of indicator. (Additional file 1 NCD Global Monitoring Framework)

Indicator category	Target indicators included (see Table 1)	# of indicators	# of indicators that need improvement						Indicator-country value	# of indicators across all countries that need improve-ment	% of the indicator-country value that needs improve-ment
			KH	LA	MY	MM	TH	VN			
1. Mortality and morbidity indicators	1–2	2	2	1	1	0	0	2	12	6	50%
2. Behavioural indicators	3–10, 15, 16	10	4	5	3	3	1	2	60	18	30%
3. Biological indicators	11–14, 17	5	1	1	0	0	0	0	30	2	7%
4. Health-service indicators based on the population surveys	18, 20–23	5	4	4	2	2	3	2	30	17	57%
5. Health-service indicators based on the facility surveys	19, 24–25	3	3	3	3	3	3	3	18	18	100%
Total	1–25	25	14	14	9	8	7	9	150	61	41%
		100%	56%	56%	36%	32%	28%	36%	100%		

KH: Cambodia, LA: Lao PDR, MY: Malaysia, MM: Myanmar, TH: Thailand, VN: Vietnam

All three-health-services indicators based on the facility survey (100%) need improvement for all six countries. These indicators include the percentage of public and private primary health-care facilities that had all the essential medicines and technologies available (Indicator #19), the percentage of health-care facilities that utilized the national guidelines for NCD management (Indicator #24), and the proportion of primary health-care facilities that offered cardiovascular risk stratification for the management of patients at high risk for heart attack and stroke (Indicator #25). The second category that most urgently required data improvement involve the health-service indicators based on the population survey. Across all six countries, 57% of the indicators require improvement. For example, all six countries lack data on the availability of palliative care for patients with NCDs in the public health system (Indicator #20). Five countries lacked data on the HPV vaccine coverage (last dose) in the previous calendar year. Three countries lacked data on the percentage of eligible persons receiving therapy and counseling to prevent heart attacks and strokes (Indicator #18). For the third category, 50% of the countries included in the indicator-country rating on the mortality and morbidity indicators need data improvement. Four countries need to improve their data on the probability of dying from NCD prematurely, and two countries need to improve their data on the estimated cancer incidence rate.

Across all six countries, the percentage of indicators that need improvements are 30% and 7% for the behavioural risk indicators and the biological risk indicators, respectively. The data availability on health risk indicators that should be improved at the country level include the mean proportion of total energy intake from saturated fatty acids (six countries) and the prevalence of alcohol use disorder/dependence (five countries).

In terms of country focus, Cambodia and Lao PDR are the two countries that need to improve their data availability on the largest number of indicators (56% of the 25 target indicators), followed by Malaysia and Vietnam (36% of the 25 target indicators). Myanmar needs to improve on 32% of indicators, and Thailand needs to improve on 28%. See Table 1 for details on indicators that need improvement.

## Discussion

Our study examined the data availability of the 25 NCDs surveillance indicators: 23 non-policy GMF indicators and 2 health service PMI indicators. We did not focus on the policy indicators of either the GMF or PMI because these indicators are easily available and reported on by each country. The *NCD Progress Monitor Report* provides complete data on these policy indicators, regardless of whether or not the policies have been implemented in the reporting countries. For the other 25 target indicators, the extent of data availability varied. The health-service indicators based on the facility survey show the highest lack of data availability, followed by the health-service indicators based on the population survey, the mortality and morbidity indicators, the behavioural risk indicators, and the biological risk indicators, respectively. This ranking of insufficient data is similar to the findings of the first global assessment of the status and capacity of health information systems in 133 countries, reported in the SCORE Global report on health data systems and capacity, 2020 [11]. This global survey found that percentages of survey-participating countries that have well developed or sustainable capacity for health service data, births/deaths/causes of death data, and populations and health risks surveys were 36%, 43%, and 54%, respectively [11].

There are two possible reasons that the biological and behavioural risk indicators are more available for the six studied countries. First, the WHO initiated the STEPwise approach to surveillance (a guide to establish risk-factor surveillance systems in countries in 2002, and 122 countries used the STEPS survey in 2012 [12]). All six of the studied countries have at least partial risk factor surveys in place [4]. Second, countries need data on NCD risk-situational factors to report on their progress in achieving the voluntary global NCD targets [2, 5, 6].

The lack of data on health-service indicators from the facility survey (Category #5) and health-service indicators from the population survey (Category #4) described in Table 2 may be because the NCD global targets give higher priority to risk-factor reductions (six out of the nine global targets) than to health-service provisions (two out of the nine targets) [2]. Moreover, the lack of adequate health information systems [13] and electronic data in health facilities [14] may contribute to the gaps in health-service indicators. To tackle NCD problems and to implement adequate and accurately targeted policies, countries need data on health-risk situational surveillance as first steps because these surveillances are cost-effective. However, in the medium term (only eight years left until 2030), implementing improvements in the availability of health services to manage NCDs along with controlling health risks (i.e., tobacco and alcohol use) are also essential in reducing NCD burdens to achieve the global targets (such as SDG target 3.4) [15]. The role of health services becomes increasingly important for short-term gains in reducing premature mortality as the deadline nears for countries to achieve their SDG goals. The reason for this is simply that impacts made by these services are much more immediate in reducing premature mortality than efforts to control NCD risk factors are. Combined with periodic population-based outcome evaluations and mapping of public health data, health-facility surveys are powerful instruments for improving health-care service delivery [16].

In addition to the lack of data availability of the crucial NCDs surveillance indicators, quality data are needed for policy, planning and patient health care design and implementation [11]. Factors influencing data quality include the representativeness of the data collected in the country, completeness of detailed indicators in each area of interest (e.g., tobacco, alcohol), and the correct definition as proposed by the WHO [17]. Furthermore, to tackle NCDs, the government needs information about the country-specific economic burden of disease and cost-effectiveness of interventions beyond these GMF and PMI indicators. However, low- and middle-income countries may lack these data as well because they do not have technically skilled health personnel for doing these kinds of analyses [18]. Another important finding was that Cambodia and Lao PDR need the biggest improvement on NCD surveillance data availability, followed by Malaysia and Vietnam. Myanmar and Thailand need to improve on some of the indicators. Country income could be one of the factors explaining the lack of health-related data availability [11]; GDP PPP per capita in 2020 rankings were from lowest to highest: Cambodia, Myanmar, Lao PDR, Vietnam, Thailand, and Malaysia [19]. Since implementing good NCD surveillance systems needs a great deal of resources, international health funding agencies and governments of low-resourced countries should recognize the necessity and invest in improving their NCD surveillance system [13].

There are a few limitations to this study. First, we addressed the data availability only, not the accuracy of the available data. Thus, this study can only recommend which indicators should be improved upon for their data availability, and cannot suggest which indicators should be improved upon for their data quality. Second, we focused our analysis only on the six selected ASEAN countries —not all 10 countries—based on the ASEAN NCD surveillance system enhancement project funded by the Thai Health Promotion Foundation, Thailand. Hence, future research should focus on all 10 ASEAN countries and be expanded to include other low- and middle-income countries (LMICs) in order to give a bigger picture to determine opportunities for improvement on the NCD surveillance system at the global level. Furthermore, studying the data quality of these NCD surveillance indicators in LMICs should also be a priority.

## Conclusion

Some of the non-policy GMF and PMI indicators lacked data among the six studied countries. Based on our findings, we recommend that countries should begin to invest in and establish the health-care facility survey and the population survey to track NCD-related health-services improvements once they have implemented the Health Risks Population Survey. Furthermore, the countries need to invest in health information systems and data collection capacity-building in order to be able to generate quality data on a regular basis. The WHO should support countries in establishing these monitoring systems for health-service indicators. Doing this would help achieve the NCD-related global targets for all the GMF and PMI indicators in the long term, not only the nine voluntary global targets. The WHO should also assist countries in collecting their own data for all indicators (and move away from using WHO estimates or expert judgments for their numbers) especially LMICs [11] because data accuracy based on their specific country contexts could improve their country’s policies and services in a more nuanced and timely manner [11].

## Electronic supplementary material

Below is the link to the electronic supplementary material.

Supplementary Material 1

## Data Availability

All data generated or analyzed during this study are included in this published article (Tables 1 and 2).

## List of Abbreviations

APC: Alcohol Consumption Per Capita
AD: Alcohol Dependence
ASEAN: Association of South-East Asian Nations
AUD: Alcohol Use Disorder
GMF: Global Monitoring Framework
HBV: Hepatitis B Virus
HED: Heavy Episodic Drinking
HPV: Human Papilloma Virus
KH: Cambodia
LA: Lao PDR
MY: Malaysia
MM: Myanmar
NCD: Non-Communicable Disease
OW/OB: Overweight and Obesity
PiA: Physical Inactivity
PMI: Progress Monitoring Indicator
SDG: Sustainable Development Goal
TH: Thailand
UN: United Nations
VN: Vietnam
WHO: World Health Organization
