# Supplementary material for: Assessing data availability of NCD prevention and control in six ASEAN countries based on WHO global monitoring framework and the progress monitor indicators
Source: BMC Public Health. 2023 Feb 7;23:272. doi: 10.1186/s12889-023-15165-1 (PMC9906914; doi:10.1186/s12889-023-15165-1)
Supplement: Supplementary file 1 — Supplementary Material 1 [file 12889_2023_15165_MOESM1_ESM.docx]

Additional file 1

NCD Global Monitoring Framework (1)

| Framework Element | Target | Indicator |  |
| --- | --- | --- | --- |
| MORTALITY & MORBIDITY | | | |
| Premature mortality from NCDs | 1. A 25% relative reduction in the overall mortality from cardiovascular diseases, cancer, diabetes, or chronic respiratory diseases | 1. Unconditional Probability of dying between ages of 30 and 70 from cardiovascular diseases, cancer, diabetes or chronic respiratory diseases |  |
| Additional indicator | | 2. Cancer incidence, by type of cancer, per 100,000 population |  |
| BEHAVIOURAL RIAK FACTORS | | | |
| Harmful use of alcohol | 2. At least 10% relative reduction in the harmful use of alcohol, as appropriate, within the national context | 3. Total (recorded and unrecorded) alcohol per capita (aged 15+ years old) consumption within a calendar year in litres of pure alcohol, as appropriate, within the national context  4. Age-standardized prevalence of heavy episodic drinking among adolescents and adults, as appropriate, within the national context  5. Alcohol-related morbidity and mortality among adolescents and adults, as appropriate, within the national context |  |
| Physical inactivity | 3. A 10% relative reduction in prevalence of insufficient physical activity | 6. Prevalence of insufficiently physically active adolescents, defined as less than 60 minutes of moderate to vigorous intensity activity daily  7. Age-standardized prevalence of insufficient physically active persons aged 18+ years (defined as less than 150 minutes of moderate-intensity activity per week, or equivalent) |  |
| Salt/sodium intake | 4. A 30% relative reduction in mean population intake of salt/sodium | 8. Age-standardized mean population intake of salt (sodium chloride) per day in grams in persons aged 18+ years |  |
| Tobacco use | 5. A 30% relative reduction in prevalence of current tobacco use in persons aged 15+ years | 9. Prevalence of current tobacco use among adolescents  10. Age-standardized prevalence of current tobacco use among persons aged 18+ years |  |
| BEHAVIOURAL RIAK FACTORS | | | |
| Raised blood pressure | 1. A 25% relative reduction in the prevalence of raised blood pressure or contain the prevalence of raised blood pressure, according to national circumstances | 11. Age-standardized prevalence of raised blood pressure among persons aged 18+ years (defined as systolic blood pressure > 140 mmHg and/or diastolic blood pressure > 90 mmHg) and mean systolic blood pressure |  |
| Diabetes and obesity | 7. Halt the rise in diabetes & obesity | 12. Age-standardized prevalence of raised blood glucose/diabetes among persons aged 18+ years (defined as fasting plasma glucose concentration > 7.0 mmol/l (126 mg/dl) or on medication for raised blood glucose)  13. Prevalence of overweight and obesity in adolescents (defined according to the WHO growth reference for school-aged children and adolescents, overweight 3 one standard deviation body mass index for age and sex, and obese – two standard deviations body mass index for age and sex)  14. Age-standardized prevalence of overweight and obesity in persons aged 18+ years (defined as body mass index > 25 kg/m^2^ for overweight and body mass index > 30 kg/m^2^ for obesity) |  |
| Additional indicator | | 15. Age-standardized mean proportion of total energy intake from saturated fatty acids in persons aged 18+ years  16. Age-standardized prevalence of persons (aged 18+ years) consuming less than five total servings (400 grams) of fruit and vegetables per day  17. Age-standardized prevalence of raised total cholesterol among presons aged 18+ years (defined as total cholesterol > 5.0 mmol/l or 190 mg/dl); and mean total cholesterol concentration |  |
| NATIONAL SYSTEMS RESPONSE | | | |
| Drug therapy to prevent heart attacks and strokes | 8. At least 50% of eligible people receive drug therapy and counselling (including glycaemic control) to prevent heart attacks and strokes | 18. Proportion of eligible persons (defined as aged 40 years and older with a 10-year cardiovascular risk > 30%, including those with existing cardiovascular disease) receiving drug therapy and counselling (including glycemic control) to prevent heart attacks and strokes |  |
| Essential noncommunicable disease medicines and basic technologies to treat major noncommunicable diseases | 9. An 80% availability of the affordable basic technologies and essential medicines, including generics, required to treat major noncommunicable diseases in both public and private facilities | 19. Availability and affordability of quality, safe and efficacious essential noncommunicable medicines, including genetics, and basic technologies in both public and private facilities |  |
| Additional indicator | | 20. Access to palliative care assessed by morphine-equivalent consumption of strong opioid analgesics (excluding methadone) per death from cancer  21. Adoption of national policies that limit saturated fatty acids and virtually eliminate partially hydrogenated vegetable oils in the food supply, as appropriate, within the national context and national programmes  22. Availability, as appropriate, if cost-effective and affordable, of vaccines against human papillomavirus, according to national programmes and policies  23. Policies to reduce the impact on children of marketing of foods and non-alcoholic beverages high in saturated fats, trans fatty acids, free sugars, or salt  24. Vaccination coverage against hepatitis B virus monitored by number of third doses of Hep-B vaccine (HepB3) administered to infants  25. Proportion of women between the ages of 30-49 screened for cervical cancer at least once, or more often, and for lower or higher age groups according to national programmes or policies |  |

Reference

1. World Health Organization. NCD Global Monitoring Framework. Geneva, Switzerland: World Health Organization; 2011. Available from: <https://www.who.int/publications/i/item/ncd-surveillance-global-monitoring-framework>. Accessed: 11/22/2022.
